# Potassium (K^+^) Starvation-Induced Oxidative Stress Triggers a General Boost of Antioxidant and NADPH-Generating Systems in the Halophyte *Cakile maritima*

**DOI:** 10.3390/antiox11020401

**Published:** 2022-02-16

**Authors:** Hayet Houmani, Ahmed Debez, Larisse de Freitas-Silva, Chedly Abdelly, José M. Palma, Francisco J. Corpas

**Affiliations:** 1 Group of Antioxidants, Free Radicals and Nitric Oxide in Biotechnology, Food and Agriculture, Department of Biochemistry, Cell and Molecular Biology of Plants, Estación Experimental del Zaidín, CSIC, Apartado 419, E-18080 Granada, Spain; houmani100@gmail.com (H.H.); larisse_bio@yahoo.com.br (L.d.F.-S.); josemanuel.palma@eez.csic.es (J.M.P.); 2 Laboratory of Extremophile Plants, Center of Biotechnology of Borj Cedria, P.O. Box 901, Hammam-Lif 2050, Tunisia; ahmed.debez@cbbc.rnrt.tn (A.D.); chedly.abdelly@cbbc.rnrt.tn (C.A.)

**Keywords:** ascorbate peroxidase, *Cakile maritima*, catalase, CuZn-SOD isozymes, halophyte, NADP-isocitrate dehydrogenase, pentose phosphate pathway, oxidative stress, potassium deficiency

## Abstract

Potassium (K^+^) is an essential macro-element for plant growth and development given its implication in major processes such as photosynthesis, osmoregulation, protein synthesis, and enzyme function. Using 30-day-old *Cakile maritima* plants as halophyte model grown under K^+^ deprivation for 15 days, it was analyzed at the biochemical level to determine the metabolism of reactive oxygen species (ROS), key photorespiratory enzymes, and the main NADPH-generating systems. K^+^ starvation-induced oxidative stress was noticed by high malondialdehyde (MDA) content associated with an increase of superoxide radical (O_2_^•−^) in leaves from K^+^-deficient plants. K^+^ shortage led to an overall increase in the activity of hydroxypyruvate reductase (HPR) and glycolate oxidase (GOX), as well as of antioxidant enzymes catalase (CAT), those of the ascorbate-glutathione cycle, peroxidase (POX), and superoxide dismutase (SOD), and the main enzymes involved in the NADPH generation in both leaves and roots. Especially remarkable was the induction of up to seven CuZn-SOD isozymes in leaves due to K^+^ deficiency. As a whole, data show that the K^+^ starvation has associated oxidative stress that boosts a biochemical response leading to a general increase of the antioxidant and NADPH-generating systems that allow the survival of the halophyte *Cakile maritima*.

## 1. Introduction

Potassium is the fourth most abundant macro-element in the lithosphere (2.5%). In a soil solution K^+^ concentration is about 0.01 to 20 mM [1], whereas this nutrient needs to be maintained within a range of 100–200 mM in the plant cell cytosol [2]. Multiple functions are ascribed to this macronutrient and have been recently reviewed [3,4,5]. K^+^ availability for plants depends on the complex dynamics of the soil which are strongly influenced by root–soil interactions [6]. In soils, K^+^ can be found as either soluble, exchangeable, fixed, or associated with minerals [7]. The release of the exchangeable form is slow and hence insufficient for plant growth and development [7]. Thus, imbalanced nutrition with K^+^ is well known. Under K^+^ limited conditions, impairment in photosynthetic apparatus occurs affecting notably the electron transfer chain and CO_2_ fixation, thus enhancing the oxygen photoreduction in chloroplasts and leading to high production of reactive oxygen species (ROS). To cope with low K^+^ availability, plants have evolved several strategies to increase K^+^ uptake and maintain their ROS homeostasis. In the few reports addressing plant response to K^+^ deficiency, the modulation of the antioxidant enzyme systems including superoxide dismutase (SOD), catalase (CAT), and all enzymatic components of the ascorbate-glutathione cycle was described [8,9,10,11]. However, the available information on the regulation of antioxidant defense upon K^+^ starvation mostly concerns glycophytes and crops, whereas halophytes remain poorly addressed. These kinds of plants are particularly challenged with the nutrient shortage in their natural habitats, whether direct (due to soil poverty) or indirect (caused by the salt-induced restriction of nutrient uptake) [12]. Thus, understanding the responses to nutrient (particularly potassium) deficiency in halophytes with emphasis on the regulation of antioxidant defense upon K^+^ starvation is pertinent, as it may enable the selection of species with high antioxidant capacity and tolerance to K^+^ deficiency.

The metabolism of ROS, which includes mechanisms of ROS generation and a set of antioxidant systems [13], keeps under control the potential ROS overproduction that can cause oxidative damages usually associated with many types of stresses, such as salinity [14], heavy metals [15,16], drought, and mechanical wounding [17]. Furthermore, NADPH is a basic indicator of cellular redox status required for cell growth, proliferation, and detoxification [18,19,20,21,22]. Thus, NADPH is strictly necessary by the antioxidant enzyme glutathione reductase (GR) in the ascorbate-glutathione pathway to regulate H_2_O_2_ content in the different subcellular compartments including cytosol, chloroplasts, mitochondria, and peroxisomes. It is also needed by the NADPH-dependent thioredoxin reductases (NTRs) in the regulation of metabolic pathways through thiol group reduction. In addition to the ferredoxin-NADP reductase (FNR) in photosynthetic cells, NADPH is mainly generated by NADP-isocitrate dehydrogenase (NADP-ICDH), NADP-malic enzyme (NADP-ME) also named NADP-malate dehydrogenase, glucose-6-phosphate dehydrogenase (G6PDH), and 6-phosphogluconate dehydrogenase (6PGDH), the latter two belonging to the oxidative part of the pentose phosphate pathway. All these enzymes are also involved in diverse, basic metabolic pathways such as carbon and nitrogen metabolisms [23,24,25,26,27].

*Cakile maritima* L. (Brassicaceae) is an annual succulent halophyte with high tolerance to osmotic constraints [28,29,30,31] which has also great potential as a nutritious crop [32]. This species is also well known for its high seed oil content (up to 40% of seed DW) [33]. The natural ecosystems for *C. maritima* are sandy littoral dunes, which are known for their poor nutrient composition [34]. Given its high nutrient use and absorption efficiencies [35], its aptitude to substitute K^+^ with Na^+^ in many biological functions, and the performance of its antioxidant system [36], *C. maritima* is a useful candidate to investigate halophyte responses to nutrient deficiencies in its antioxidant response. Therefore, the present study aimed at better characterizing the response of *C. maritima* to K^+^ deficiency with special emphasis on the plant growth, the water content, the antioxidative stress response, and the nutrient status.

Overall, the data provide evidence that K^+^ starvation provoked oxidative stress which triggers a general boost of the main antioxidant systems, and this was also accompanied by an increase in the NADPH-generating system in both roots and leaves, thus allowing the survival of *C. maritima*.

## 2. Materials and Methods

### 2.1. Plant Material and Growth Conditions

**Figure 1:**
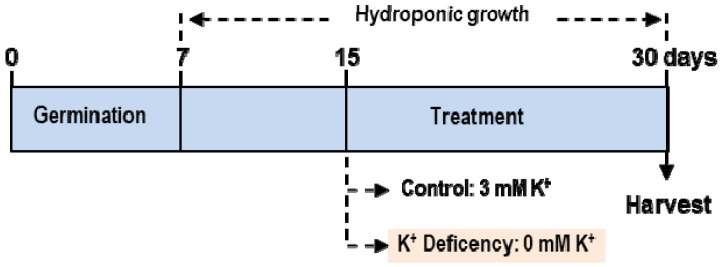
Experimental design to study potassium deficiency in the halophyte *Cakile maritima* L. Seeds were germinated in Petri dishes for 7 days. Then, the plantlets were hydroponically grown in half-strength Hoagland’s nutrient solution for an additional 7 days. Thereafter, plants were separated in two lots: control (C) plants kept in the Hoagland nutrient solution containing 3 mM K^+^ and deficient plants, cultivated after transplanting to a medium without K^+^. After 15 additional days, plants were harvested.

*C. maritima* seeds were cleaned with commercial sodium hypochlorite (50%; *w/v*) for 4 min. Then they were washed five times with distilled water, and then sown in foil-covered Petri dishes containing two layers of filter paper imbibed with 15 mL H_2_O [28]. After seven days, plantlets were hydroponically grown in half-strength Hoagland’s nutrient solution (2.5 mM Ca(NO_3_)_2_·4H_2_O, 2.5 mM KNO_3_, 0.5 mM KH_2_PO_4_, and 1 mM MgSO_4_·7H_2_O for the macronutrients; and 23.2 μM H_3_BO_3_, 4.6 μM MnCl_2_·4H_2_O, 1.2 μM ZnSO_4_·7H_2_O, 0.185 μM CuSO_4_·5H_2_O, and 0.06 μM Na_2_MoO_4_·2H_2_O for the micronutrients) for an additional 7 days. Thereafter, plants were separated into two sets to study K^+^ deficiency: control plants were kept in the Hoagland nutrient solution, and deficient plants were cultivated after transplanting to a medium without K^+^. The deficiency of NO_3_^−^ and PO_4_^3−^ in the nutrient solution was corrected by the addition of appropriate amounts of NaNO_3_ and (NH_4_)H_2_PO_4_ [10,37]. The plant culture was carried out under greenhouse conditions (16 h photoperiod, 24/18 °C light/dark temperature; 80% relative humidity). After another 15 days, plants were harvested and separated into roots and leaves and stored at −80 °C for biochemical assays. Figure 1 shows the experimental design to study K^+^ deficiency in *C. maritima.*

### 2.2. Plant Crude Extracts

Plant organs (roots and leaves) were collected and frozen in liquid N_2_. Then, the samples were ground to a powder in a mortar with a pestle. Two grams of the powder were suspended in a ratio 1/2 (*w/v*) in 50 mM Tris-HCl buffer (pH 7.8) containing 0.1 mM EDTA, 0.02% (*w/v*) Triton X-100, 10% (*v/v*) glycerol, 1% (*w/v*) polyvinylpolypyrrolidone (PVPP), and 5 mM dithiothreitol (DTT). The crude extracts were then filtered through one layer of Miracloth and centrifuged at 27,000× *g* at 4 °C for 25 min. Finally, the supernatants were collected and used for assays.

### 2.3. Water Content

Water content (WC) of each organ was calculated as the difference between the fresh (FW) and dry (DW) weights according to the following equation WC = (FW − DW)/DW. Plant material was dried for 5 days at 65 °C on a stove.

### 2.4. Potassium Content and Cellular Potassium Concentration

To determine K content in leaves and roots, samples were oven-dried at 60 °C and then ground into fine powder. K was extracted from the obtained powder (approximately 30–60 mg) by digestion with nitric/perchloric acid, and K analysis was performed using atomic absorption spectrophotometry (Perkin Elmer 1100 B). Cellular K concentration, a crucial parameter for the evaluation of K^+^ status inside the plant cell, was estimated as the ratio of K^+^ and water contents.

### 2.5. Lipid Peroxidation and Histochemical Detection of Superoxide Radical (O_2_^•−^) in Leaves

Lipid peroxidation products were estimated by measuring the malondialdehyde (MDA) content through the thiobarbituric acid reactive substances (TBARS) method [38].

For the histochemical detection of O_2_^•−^, leaves from control and K^+^-deficient plants were excised and vacuum-infiltrated for 5 min in a nitroblue tetrazolium (NBT) solution (0.5 mg mL^−1^ in 100 mM phosphate buffer, pH 6.8). After infiltration, the samples were incubated for 1 h at 25 °C in darkness. Then, leaves were illuminated until the appearance of dark blue spots, characteristic of blue formazan precipitates [39].

### 2.6. Anthocyanins

Anthocyanin content was determined according to the Gould et al. (2000) [40] method. Samples were preserved in 2 mL of a solution containing (HCl/H_2_O/methanol) (*v/v/v*; 1/3/6) and stored at 4 °C in the dark until the subsequent pigment extraction. The absorbance was read at 530 and 653 nm. The following formula was used to determine the anthocyanin content:

Anthocyanins concentration (μg mL^−1^) = OD_530_ − 0.24·OD_653_

### 2.7. In-Gel Isozyme Profile Analyses of Superoxide Dismutase (SOD), Peroxidase (POX), and Ascorbate Peroxidase (APX)

The SOD isozymes were separated by non-denaturing polyacrylamide gel electrophoresis (PAGE) on 8% acrylamide gels and visualized by a photochemical NBT reduction method [41]. The type of SOD isozymes was identified according to its sensitivity to different inhibitors, 5 mM KCN or 5 mM H_2_O_2_. CuZn-SOD is inhibited by KCN and H_2_O_2_; Fe-SOD is only inhibited by H_2_O_2_ while Mn-SOD is unaffected by either KCN or H_2_O_2_ [28]. The POX isozymes were separated by non-denaturing PAGE on 6% acrylamide gels and detected as previously described by [42]. Briefly, gels were incubated for 20 min in sodium acetate buffer 0.1 M, pH 5.5 containing 3,3-diaminobenzidine 1 mM, and H_2_O_2_ (0.03%), and brown bands appeared at the end of the reaction. APX isozymes were separated as described by [43]. Briefly, gels were prepared at 10% acrylamide and run for 30 min at 120 V before samples were loaded. Electrophoresis was conducted for 3 h (120 V, 4 °C). Then, gels were incubated three times in 50 mM potassium phosphate buffer, pH 7.0, containing 2 mM ascorbic acid for 10 min each, and once in 50 mM potassium phosphate buffer, pH 7.0, containing 4 mM ascorbic acid and 0.5 μM H_2_O_2_ for 10 min. After the last incubation, gels were rinsed twice with distilled water and equilibrated for 1–2 min in 50 mM potassium phosphate buffer pH 7.8. The staining reaction was started by adding 50 mM potassium phosphate buffer pH 7.8 containing 14 mM TEMED and 2.45 mM NBT. The reaction was stopped when the first blue bands became visible by decanting the staining solution and rinsing the gels with distilled water.

### 2.8. Determination of Enzyme Activities

Glycolate oxidase (GOX; EC 1.1.3.1) was assayed by determining the formation of the complex glyoxylate-phenylhydrazone as described previously by [44]. NADH-dependent hydroxypyruvate reductase (HPR; EC 1.1.1.2 9) was assayed according to Schwitzguébel and Siegenthaler (1984). Catalase activity (CAT; EC 1.11.1.6) was determined using the [45] method which consists of measuring the disappearance of H_2_O_2_ at 240 nm. Ascorbate peroxidase (APX; EC 1.11.1.11) was determined by monitoring the initial ascorbate oxidation by H_2_O_2_ at 290 nm [46]. Monodehydroascorbate reductase (MDAR; EC 1.6.5.4) was assayed by measuring the monodehydroascorbate-dependent NADH oxidation, with monodehydroascorbate being generated by the ascorbate/ascorbate oxidase system [47]. The rate of monodehydroascorbate-independent NADH oxidation (without ascorbate and ascorbate oxidase) was subtracted from the monodehydroascorbate-dependent reaction. Glutathione reductase (GR; EC 1.6.4.2) was measured following the Edwards et al. (1990) [48] method by monitoring the NADPH oxidation at 340 nm coupled to the reduction of GSH (the reaction rate was corrected for the small, non-enzymatic oxidation of NADPH by glutathione disulfide, GSSG). Dehydroascorbate reductase (DHAR; EC 1.8.5.1) was determined by following the increase of ascorbate formation at 265 nm using N_2_-saturated buffer [49]. The reaction rate was corrected by the non-enzymatic reduction of dehydroascorbate by glutathione (GSH). A factor of 0.98, to account for the small contribution to the absorbance by GSSG, was also considered.

NADP-dependent dehydrogenase (NADP-DH) activities were determined spectrophotometrically by recording the reduction of NADP^+^ at 340 nm. The assays were performed at 25 °C in a reaction medium (1 mL) containing 50 mM HEPES, pH 7.6, 2 mM MgCl_2_, and 0.8 mM NADP. The reaction was initiated by the addition of a specific substrate for each enzyme. Thus, NADP-ICDH (EC 1.1.1.42) activity was started by the addition of 10 mM 2R,3S-isocitrate; G6PDH (EC 1.1.1.49) activity was initiated by the addition of 5 mM glucose-6-phosphate; to determine 6PGDH (EC 1.1.1.44) activity, the substrate was 5 mM 6-phosphogluconate was initiated; and, in the case of NADP-ME (EC 1.1.1.40) activity, the reaction was started by the addition of 1 mM L-malate [50,51,52]. Protein concentration was determined using the Bio-Rad protein assay with bovine serum albumin as standard.

### 2.9. Statistical Analysis

Statistical analysis was performed using the Statgraphics program and data were analyzed by one-way ANOVA. Asterisk denotes that means are significantly different at *p* < 0.05.

## 3. Results

### 3.1. Effect of Potassium Starvation on C. maritima Growth Parameters

To corroborate the potassium deficiency in the experimental design, the potassium content in roots and shoots was evaluated. Table 1 shows that in *C. maritima* plants grown in nutrient solutions deficient in potassium, the K^+^ content and concentration were significantly lower in both organs (on average 86%) in comparison to plants grown under optimal conditions.

**Figure 2:**
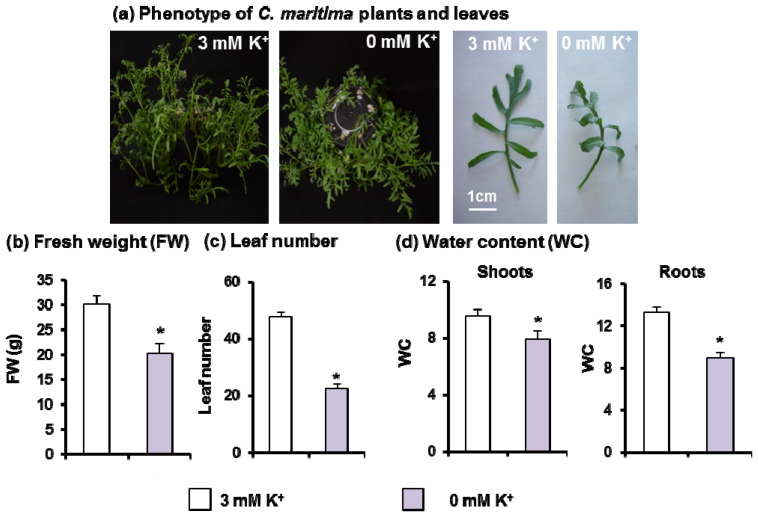
Phenotype and growth attributes of 30-day-old *C. maritima* plants grown in the presence or absence of K^+^ in the culture medium for 15 days. (**a**) Phenotype of *C. maritima* growth in the hydroponic cultivation and leaf detail. (**b**) Plant fresh weight (FW). (**c**) Leaf number per plant. (**d**) Shoot and root water contents (WC). Results are the mean of at least three different experiments ± SEM. Asterisks indicate that differences between values were statistically significant at *p* < 0.05.

Figure 2a shows a representative picture of 30-day-old *C. maritima* plants grown under potassium deficiency (0 mM K^+^) which showed a 33% decrease in plant biomass production (Figure 2b) as compared to plants cultivated on optimal conditions (3 mM K^+^). A 53% reduction in leaf number was also observed under the same conditions (Figure 2c). The lack of K^+^ in the culture medium negatively affected both leaf and root water content (17% and 30%, respectively), suggesting the induction of water stress by K^+^ deficiency (Figure 2d).

### 3.2. Metabolism of ROS and Photorespiration under K^+^ Deficiency in C. maritima

**Figure 3:**
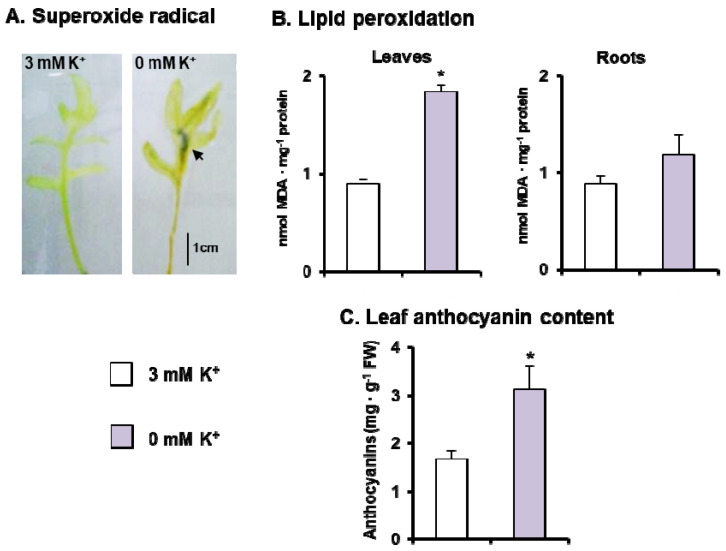
ROS parameters and anthocyanin content in 30-day-old *C. maritima* plants grown in the presence or absence of K^+^ in the culture medium for 15 days. (**A**) Histochemical detection of superoxide radical with NBT staining in leaves. Arrow indicates the precipitated blue formazan product. (**B**) Lipid peroxidation (MDA) in leaves and roots. (**C**) Leaf anthocyanin content. Results are the mean of at least three different experiments ± SEM. Asterisks indicate that differences between values were statistically significant at *p* < 0.05.

Lipid peroxidation, as a marker of membrane oxidative damages, and production of O_2_^•−^ were used to evaluate the impact of ROS metabolism under K^+^ deficiency. The in vivo production of O_2_^•−^ was assayed as the reduction and further precipitation of NBT leading to the appearance of dark spots of blue formazan in leaves from *C. maritima* plants subjected to K^+^ deficiency, but not in leaves from control plants (Figure 3A). As a potential result of the accumulation of O_2_^•−^ and dismutation to H_2_O_2_, oxidative damage to membrane lipids was investigated by evaluating the MDA content, the final product of lipid peroxidation. There was a significant increase in MDA content (49%) in leaves from plants grown under K^+^ deficiency; in roots, the MDA values remained close to the control (Figure 3B). On the other hand, the content of anthocyanins increased about 53% in leaves of *C. maritima* plants grown under K^+^ deficiency (Figure 3C).

**Figure 4:**
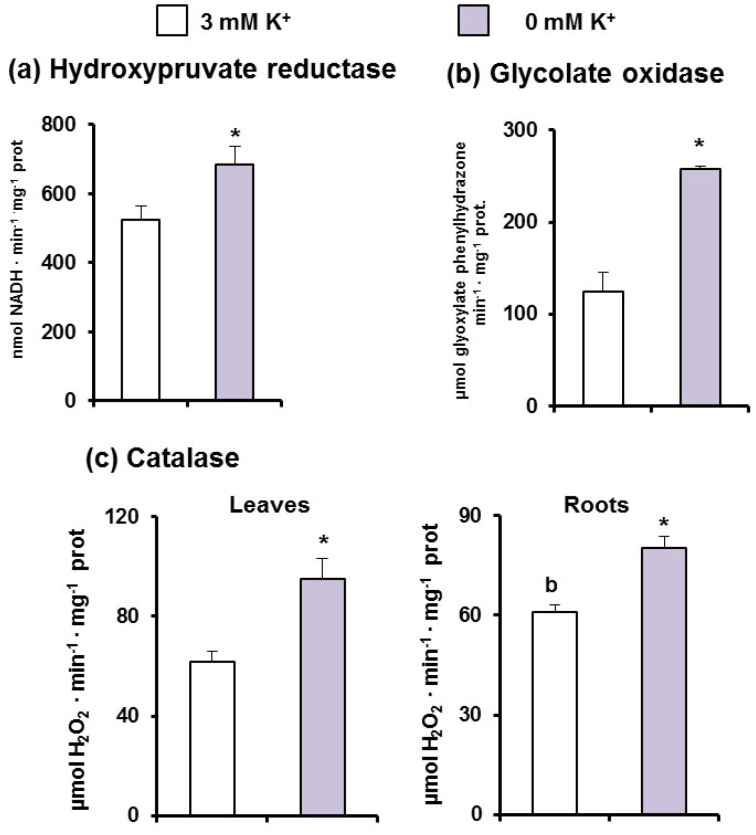
Activities of photorespiratoy enzymes and catalase in *C. maritima* plants grown in the presence or absence of K^+^ in the culture medium for 15 days. (**a**) Glycolate oxidase activity. (**b**) Hydroxypruvate reductase activity. (**c**) Catalase activity. Results are the mean of three different experiments ± SEM. Asterisks indicate that differences between values were statistically significant at *p* < 0.05.

Two peroxisomal photorespiratory enzymes were also analyzed. Thus, whereas hydroxypyruvate reductase (HPR) grew about 20% (Figure 4a), glycolate oxidase (GOX), which is considered one of the main sources of H_2_O_2_ in green tissues, increased 2.8-fold (Figure 4b) under K^+^ starvation. On the other hand, catalase, the main H_2_O_2_-removing enzyme also located in peroxisomes, increased 1.8-fold in leaves and 1.3-fold in roots of plants grown under K^+^ deficiency (Figure 4c).

**Figure 5:**
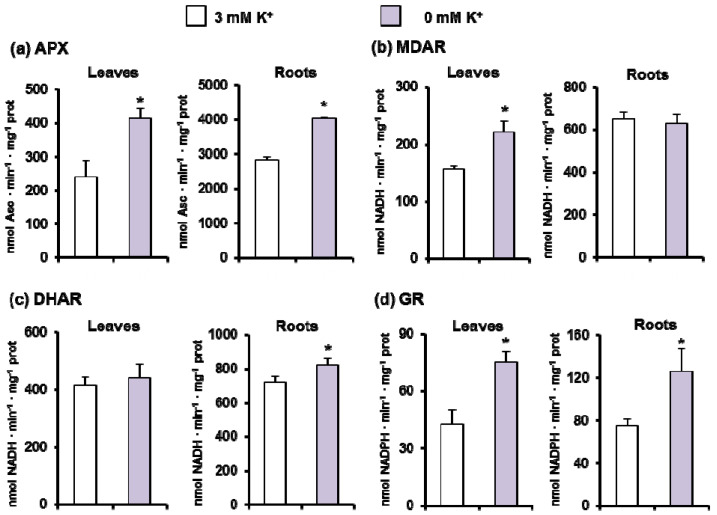
Ascorbate-glutathione cycle activities in leaves and roots of 30-day-old *C. maritima* plants grown in the presence or absence of K^+^ in the culture medium for 15 days. (**a**) Ascorbate peroxidase (APX) activity. (**b**) Monodehydroascrobate reductase (MDAR) activity. (**c**) Dehydroascorbate reductase (DHAR) activity. (**d**) Glutathione reductase (GR) activity. Results are the mean of three different experiments ± SEM. Asterisks indicate that differences between values were statistically significant at *p* < 0.05.

**Figure 6:**
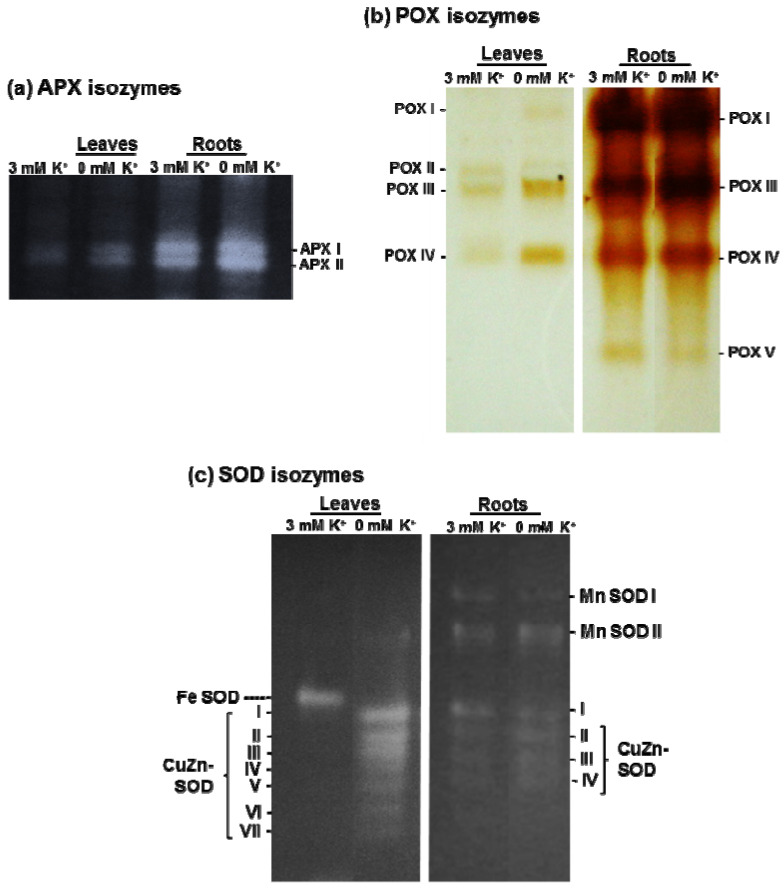
Analysis of APX, POX, and SOD isozymes in *C. maritima* plants grown in the presence or absence of K^+^ in the culture medium for 15 days. (**a**) Ascorbate peroxidase (APX) isozymes (40 μg of proteins per lane). (**b**) Peroxidase (POX) isozymes (80 μg of proteins per lane). (**c**) Superoxide dismutase (SOD) isozymes (100 and 40 μg of proteins per lane were used for leaves and roots, respectively). The different isozymes were separated by native PAGE (8% for SOD, 10% for APX, and 6% for POX).

Given the importance of the ascorbate-glutathione cycle to control the cellular H_2_O_2_ content in coordination with catalase, the activity of its enzymatic components (APX, MDAR, DHAR, and GR) was spectrophotometrically assessed (Figure 5a–d). In general, the activity of these enzymes was higher in roots than in leaves, and under K^+^ deficiency a significant increase of all these activities in both organs was found, except for MDAR in roots and DHAR in leaves that were unaffected. The data on APX activity correlates with the isozyme pattern obtained after native PAGE and specific in-gel activity staining. Two APX isozymes were detected in leaves and roots, being the most prominent isozymes in roots. Under K^+^ deficiency, there was a slight increase of APX isozymes in both organs (Figure 6a).

The analysis of POX isozyme patterns in *C. maritima* revealed essentially the same profile in leaves and roots, with a total of five isozymes designated I to V according to their increased mobility in the gel, with the intensity of the bands considerably higher in roots than in leaves (Figure 6b). Under K^+^ starvation, in leaves POX I, III, and IV increased. However, in roots there were no apparent changes due to K^+^ deficiency, except for a slight decrease of POX V, an isozyme that was not detected in leaves.

Figure 6c shows the analysis of SOD isozyme activity patterns obtained after native PAGE and NBT staining of gels in both leaves and roots from *C. maritima* grown under optimal and low K^+^ supply. In leaves from control plants, only a single Fe-SOD was present in crude extracts. However, under K^+^ deficiency this isozyme was undetectable, whereas until seven CuZn-SODs isozymes (designated as I to VII) were induced, CuZn-SODs I to III were the most prominent. In roots, two Mn-SOD and four CuZn-SOD (I to IV) isozymes were identified, with a light activity increase under K deficiency (Figure 6c).

### 3.3. Metabolism of NADP-Dehydrogenases under K^+^ Deficiency in C. maritima

**Figure 7:**
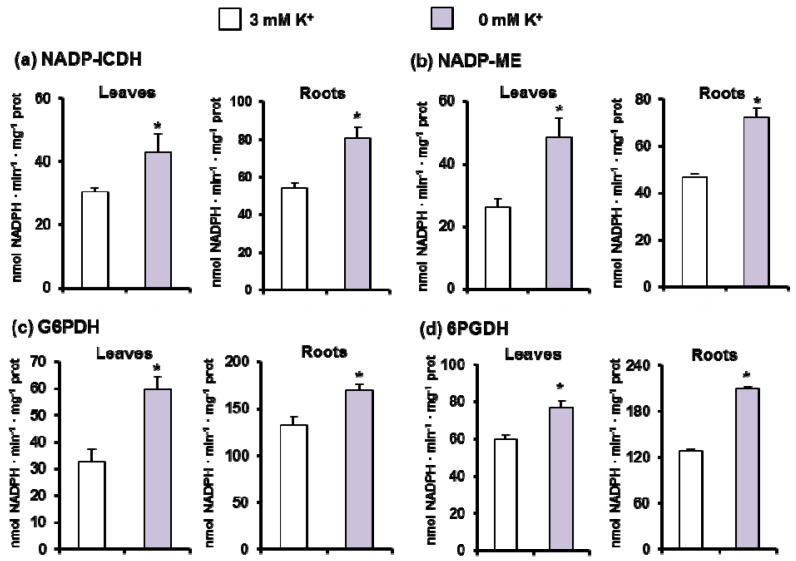
NADP-dehydrogenase activities in leaves and roots of 30-day-old *C. maritima* plants grown in the presence or absence of K^+^ in the culture medium for 15 days. (**a**) NADP-isocitrate dehydrogenase (ICDH) activity. (**b**) NADP-malic enzyme (ME) activity. (**c**) Glucose-6-phosphate dehydrogenase (G6PDH) activity. (**d**) 6-phosphogluconate dehydrogenase activity (6PGDH). Data represent the mean ± SEM of at least three different experiments. Asterisks indicate that differences between values were statistically significant at *p* < 0.05.

Figure 7 illustrates the activity of the four main NADPH-generating enzymes, NADP-ICDH (Figure 7a), NADP-ME (Figure 7b), and the two enzymes of the oxidative pentose phosphate pathway, G6PDH and 6PGDH (Figure 7c,d, respectively). In general, the activity of these NADP-dehydrogenases was higher in roots than in leaves, and under K^+^ deficiency all activities underwent a significant increase in both organs.

## 4. Discussion

### 4.1. K^+^ Deficiency Alters C. maritima Growth and Induces Oxidative Stress

K^+^ deficiency disturbs water status in leaves and roots of *C. maritima*, suggesting osmotic stress caused by the lack of this element in the medium. The root is the first organ that senses K^+^ deficiency and consequently, a series of responses occur at physiological, biochemical, and molecular levels. According to [53], at an earlier phase of K^+^ deficiency exposure, cytosolic K^+^ content in plant tissue is regarded as one of the “master switches” which is responsible for plant transition from the ordinary metabolism to a “hibernated state”. In *C. maritima* roots, water stress could be explained by the fact that plant K^+^ status affects greatly the activity of aquaporins, as revealed by [54] who demonstrated that the activity of aquaporins in tomatoes was suppressed under low K^+^ supply and was associated with a decrease of water transport to shoots. Given its implication in osmotic adjustments, K^+^ deficiency is known to disturb transpiration causing a severe inhibition of leaf expansion [55], supporting our findings on leaves of *C. maritima* subjected to K^+^ starvation.

K^+^ is implicated in the regulation of guard cells during stomatal movements and its deficiency leads to stomatal closure. Such behavior was documented in sunflowers and olives, in which the K^+^ status greatly affected stomata closure level [56]. This condition leads to an inhibition of photosynthesis rate, transpiration, stomatal conductance, and disturbing plant water relations [57] and, as a consequence, an imbalance between photosynthetic CO_2_ fixation and excessive accumulation of non-scavenged electrons may provoke the generation of reactive oxygen species (ROS) [58,59]. In *Arabidopsis thaliana* it was found that the loss of function of the chloroplast K^+^ Efflux Antiporters KEA1 and KEA2, located in the inner envelope membrane, provoked inefficient photosynthesis [60] and altered ROS homeostasis in leaves and roots [61]. It was reported that in plants K^+^ deficiency may act as a stress signal, since K^+^ fluxes in the plants affect the normal functioning of many metabolic processes as well as ROS homeostasis [3,62,63], and may hence induce many adaptive responses to withstand the lack of K^+^ in the medium. K^+^ must trigger cellular signaling alone or in association with other signaling molecules and phytohormones [3,5]. In our case, the estimation of the cellular K^+^ concentration, a crucial parameter for the evaluation of K^+^ status inside the plant cell, demonstrated a significant decrease upon K^+^ deprivation in both leaves and roots. Indeed, cytosolic K^+^ concentration is considered as a signal that affects several metabolic pathways including ROS metabolism. As described by [64], changes in K^+^ status during the first hours of K^+^ starvation resulted in several responses, particularly the ROS metabolism. Furthermore, it has been established that the decrease in cytosolic K^+^ concentration under long-term K^+^ deficiency provoked many morphological and metabolic changes including ROS generation [2]. Taken together, the disruption in plant water relations and the decrease in intracellular K^+^ concentration explain the K^+^ deficiency-induced oxidative stress in *C. maritma*.

It is known that at high levels, superoxide radical (O_2_^•−^) disrupts the normal functioning of plant cells and, in our experimental conditions, this ROS was accumulated in K^+^-deficient leaves. A similar increase of O_2_^•−^ and H_2_O_2_ contents was found in maize subjected to K^+^ deprivation associated with chloroplast damages [65]. Likewise, K^+^ deficiency increased ROS content in tomato and cotton roots [66,67], soybean, and maize leaves [68]. Like other free radicals, O_2_^•−^ may directly or indirectly negatively affect proteins and nucleic acids (DNA and RNA) and cause disruptions to membranes [69,70]. In this context, the analysis of lipid peroxidation showed that in plants grown in K^+^-deficient medium, MDA content was significantly higher in *C. maritima* leaves compared to the control. A comparable finding has been described in other plant species [66,68]. It has been documented that plant exposure to nutrient deficiency stress is associated with a higher production of free radicals and significant damages to lipid membranes [71], and this depends on the plant species and genotype, as observed in soybean [71] and maize [65].

The increased lipid peroxidation could be due in part to the accumulation of O_2_^•−^ and its dismutation to H_2_O_2_, but also to the increased photorespiratory H_2_O_2_ production, resulting from the enhancement of GOX activity in leaf peroxisomes from plants suffering K^+^ deficiency. GOX is very abundant in photosynthetic tissues of C_3_ plants where it could represent about 1% of total proteins [72], it has been documented that photorespiration is among the photoprotective mechanisms against oxidative stress when an imbalance in photosynthesis light-harvesting and utilization occurred [73]. Similar findings were obtained by Singh and Blanke (2000) [74], who demonstrated the stimulation of photorespiration under K^+^ shortage conditions. Anthocyanins provide a purple/red appearance of leaves, and they have a photoprotective function [75,76]. Our experimental conditions of K^+^ deficiency triggered a rise of 53% on the anthocyanin content, which is in good agreement with the general increase of antioxidant systems.

### 4.2. Oxidative Stress Is Overcome by a General Induction of the Enzymatic Antioxidant System

Plants respond to ROS excess through the coordination of complex antioxidant systems including both non-enzymatic (ascorbic acid and glutathione, among others) and enzymatic antioxidants located in different cellular compartments, mainly catalase, SOD, and the ascorbate-glutathione cycle. The role of those antioxidants is to keep ROS homeostasis locally in the cytosol, chloroplasts, peroxisomes, and mitochondria, but also globally within the cell [77,78,79].

Given the high antioxidant capacity developed by the halophyte *C. maritima* under various abiotic stresses [17,28,36], we hypothesized a stimulation of its antioxidant systems under K^+^ shortage. The induction of up to seven CuZn-SOD isozymes in leaves under K^+^ deficiency conditions was outstanding. Our results are also in good agreement with those from Liu et al. (2013) [80] and Li et al. (2020) [11] who demonstrated an increase in total SOD activity in rice and alligator weed plants subjected to K^+^ deficiency for 12 and 15 days, respectively. The inducible CuZn-SODs found in this work were reported to play a primordial role in *C. maritima* tolerance to various abiotic stress including salinity [28] and mechanical wounding [17]. In those studies, we demonstrated that *C. maritima* contains a total of ten SOD isozymes: two Mn-SODs, one Fe-SOD, and seven CuZn-SODs, and that CuZn-SODs were the main isozymes modulated under severe long-term salinity stress. Thus, it is not surprising that these later isozymes might be induced by other abiotic stresses like K^+^ deficiency. A new CuZn-SOD isozyme was induced by Mg^2+^ deficiency in cotton plants [81], thus suggesting that the change of the intensity of existing SOD isozymes or the induction of new ones are among the most adaptive traits to Mg^2+^ deficiency-induced oxidative stress in *Gossypium hirsutum*. Likewise, in tomato roots under salinity stress and treated with a nitric oxide donor, it was found that there was an induction of the CuZn-SOD (III) in roots, suggesting that nitric oxide could exert a regulation at the protein and gene level of this antioxidant enzyme [82], being a plausible issue in *C. maritima* that deserves to be explored.

H_2_O_2_ generated by the enhanced activity of SOD under K^+^ starvation may also stimulate other antioxidant components in *C. maritima* under such conditions. In this regard, H_2_O_2_ is recognized as a key signaling molecule [83] and, under K^+^ deficiency, a general increase of the most relevant antioxidant systems that control H_2_O_2_ cellular content such as catalase, located in peroxisomes [84], and all the enzymes of the ascorbate-glutathione cycle in leaves and roots of *C. maritima* plants was observed. These findings support previous reports indicating that the lack of K^+^ in the medium affects ROS metabolism in different plant species. It has been pointed out that short (one–seven days) or long-term (14–60 days) K^+^ starvation differently affected the antioxidant response in plants [6,66,68]. According to the literature, under low K^+^ supply catalase activity was enhanced, unaffected, or diminished and was modulated depending on the plant organ. In rice [85] and *Brassica juncea* [86], K^+^ deprivation led to an increase in catalase activity, whereas a decrease of this activity was observed in *Solanum lycopersicum* roots [66].

On the other hand, peroxidases (POX) ensure multiple biological processes in plant cells. Our data indicated that an overall increase of POX activity takes place in *C. maritima* due to K^+^ shortage. Hafsi et al. (2011) [8] also demonstrated an induction of guaiacol peroxidase activity in wheat subjected to K^+^ starvation. The sensitive genotype of potato grown under K^+^ deficiency also showed the highest CAT and POX activities in roots compared to the tolerant one [10]. A modulation of POX activity was observed as well upon K^+^ deficiency, as revealed by transcription analysis in rice [87]. Recently, proteomic studies of the antioxidant enzymes showed changes in abundance of SOD, POX, glutathione-*S*-transferase (GST), and glutathione peroxidase (GPX) in stems of alligator weed (*Alternanthera philoxeroides*) plants under K^+^-deficiency stress [88]. More recently, quantitative proteomic analysis of alligator weed leaves demonstrated an increase in abundance of cationic peroxidase 1 (ApCPX1) which played a relevant role when plants underwent K^+^ deficiency stress [11].

Specific ascorbate peroxidase (APX) isozyme activities, analyzed by spectrophotometric assay and native PAGE, were increased in leaves and roots from K^+^-deficient *C. maritima* plants. Liu et al. (2013) [80] and Ahmad et al. (2014) [86] reported an enhancement of APX activity in rice and *B. juncea* under limiting K^+^ supply. Such results provide evidence for the important role of APX in H_2_O_2_ detoxification in the different organelles under unfavorable conditions [89,90]. At the molecular level, several studies have revealed an upregulation of genes encoding peroxidases in *A. thaliana* [91] and *S. lycopersicum* [66]. APX and other peroxidases are good ROS scavengers that may also operate at the apoplast together with CuZn-SODs, thus contributing to mitigate the harmful effects of oxidative stress.

Several enzymes of the ascorbate-glutathione cycle are implicated in H_2_O_2_ detoxification: APX, MDAR, DHAR, and GR. A stimulation of GR, MDAR, and DHAR activities was noted under K^+^ deficiency in *C. maritima*. Hafsi et al. (2011) [8] found an increase of DHAR and MDAR activities in *Hordeum maritimum* exposed to K^+^ deprivation and attributed such results to the fact that both enzymes generate ascorbate implicated in H_2_O_2_ detoxification. K^+^ deficiency also enhanced the activity of GR in rice leaves [80] and cotton roots [67]. This enzyme reduces oxidized glutathione (GSSG) to GSH, a tripeptide that is involved in antioxidant processes under stressful conditions [92].

ROS metabolism plays a key role in the acclimation process of plants to abiotic stress. H_2_O_2_ produced through the activity of GOX, SOD, and perhaps other enzymes can be used as a signaling molecule that plays an important role in *C. maritima* acclimation to K^+^ deficiency. Many studies highlight the ability of plants to use ROS in many signaling pathways [93,94,95,96]. ROS is involved in the signaling cascade that induces the expression of genes coding for HAK5, a high-affinity K^+^ transporter [97]. Furthermore, it has been reported that, under K^+^ deficiency conditions, the interplay between ROS accumulation and increased ethylene production is a key in signaling K^+^ root status and activating many morphological adaptations to K^+^ deficiency, such as primary root growth, root hair elongation, and induction of HAK5, resulting in an enhancement of K^+^ uptake and plant survival under K^+^ limiting conditions [98,99]. Moreover, overexpression of peroxidase in *Arabidopsis thaliana* has been discussed to improve *AtHAK5* expression in response to K^+^ shortage conditions through its contribution to ROS production [100].

### 4.3. K^+^ Deficiency Triggers a General Increase of the NADPH-Generating Systems

For a deeper knowledge about the likely role of NADPH as a second line of defense to support some of the antioxidant systems [17] in *C. maritima* under K^+^ shortage and given the lack of information on NADPH implication in plant response to K^+^ deficiency in other species, we assessed the activity of four main enzymes responsible for its generation. Our results show that the activity of NADP-ICDH, NADP-ME, G6PDH, and 6PGDH increased by low K^+^ availability in leaves and roots. The stimulation of NADPH-generating enzymes has been described under various abiotic stresses including salinity, heavy metal stress, and drought [16,18,21,51,101,102,103,104,105,106]. For example, under chromium stress, all NADP-DHs were increased in roots and coleoptile of maize seedlings [15] and in *A. thaliana*, 20 µM glyphosate stimulated the activity of G6PDH and 6PGDH [20]. More recently, Houmani et al. (2018) [17] demonstrated an increase of NADP-ICDH under mechanical wounding in seven-day-old *C. maritima* seedlings. Using quantitative proteomic analyses, Li et al. (2018) [107] demonstrated downregulation of NADP-ICDH abundance upon K^+^ deprivation in roots of *Alternanthera philoxeroides* and they explained such results by the fact that this enzyme is responsible for citric acid accumulation which in turn is secreted into the soil solution to dissolve K^+^ and facilitate its absorption at the root plasma membrane. Globally, all these data support the relevance of the NADPH-generating systems under adverse environmental conditions and specifically in the K^+^ nutrition in plants.

## 5. Conclusions

**Figure 8:**
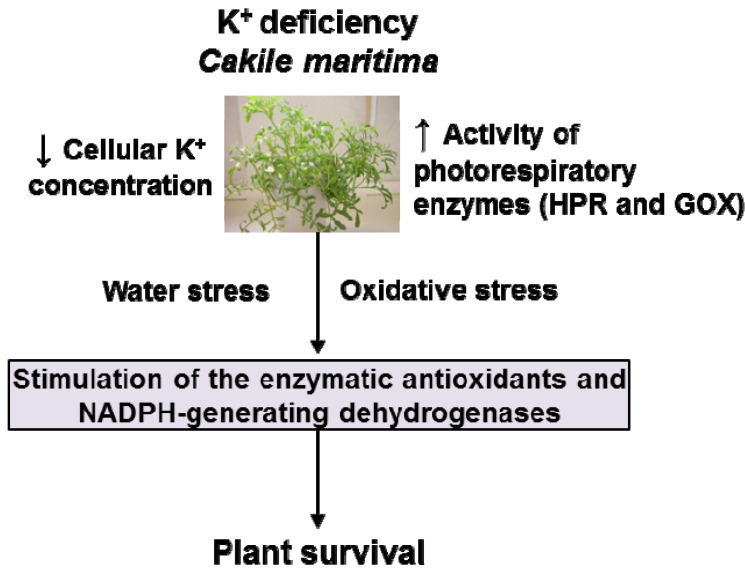
Model of the mechanism of response of halophyte *C. maritima* grown under K^+^ deficiency. GOX, glycolate oxidase. HPR, hydroxypyruvate reductase.

All the obtained biochemical results indicate that under low K^+^ availability, *C. maritima* plants undergo oxidative stress. This event triggers a general boost of the antioxidant systems, particularly the induction of up to seven new CuZn-SOD isozymes. Likewise, K^+^ starvation induces a generalized increase in the enzymes responsible for generating reducing power in the form of NADPH, which contributes to maintaining the cellular redox homeostasis. All these biochemical mechanisms allow *C. maritima* to survive under K^+^ starvation, conditions that do not allow the survival of other plant species (Figure 8). Considering that some terrestrial areas undergo a growing deterioration in the quality of the land due to a lack of nutrients such as K^+^, or the increasing soil salinization where glycophytic plants cannot survive, *C. maritima* should be considered an alternative crop for this type of soil. Furthermore, *C. maritima* is a halophyte plant that has been used for different purposes, such as culinary uses and as a dried ingredient in flours to make bread or flavoring for salads; and its seeds are also appreciated for its oils which constitute around 40% of its dry weight [32,33]. Likewise, another industrial potential use could be for its high content of antioxidant enzymes such as the CuZn-SOD, which is induced under K^+^ availability (the present study) or high salinity [28]. These potentialities could be explored due to further applications, either as a nutraceutical complement or for therapeutic/industrial use since antioxidant enzymes are emerging components for the pool of industrial enzymes.

## Figures and Tables

**Table 1:** K^+^ content and K^+^ concentration (mg mL^−1^) in shoots and roots of *C. maritima* under optimal or limiting K^+^ conditions. Results are the mean of at least three different experiments ± SEM. Asterisk indicates that values in the same row are significantly different at *p* < 0.05.

Organs	3 mM K^+^	0 mM K^+^
**Shoot K content** (mg g^−1^ dry weight)	4.05 ± 0.14	0.46 ± 0.04 *
**Root K content** (mg g^−1^ dry weight)	3.89 ± 0.57	0.56 ± 0.09 *
**Shoot K concentration** (mg mL^−1^)	0.43 ± 0.02	0.065 ± 0.003 *
**Root K concentration** (mg mL^−1^)	0.29 ± 0.02	0.063 ± 0.010 *

## Data Availability

Data is contained within the article.
